# Swedish Normative Scores for the BREAST-Q Reduction/Mastopexy Module

**DOI:** 10.1007/s00266-022-03025-z

**Published:** 2022-08-03

**Authors:** Salma Tunå Butt, Emmelie Widmark-Jensen, Susanne Meyer, Emma Hansson

**Affiliations:** 1 Institution of Clinical Sciences, Departement of Surgery, Skåne University Hospital, Lund University, Malmö, Sweden; 2 Institute of Clinical Sciences, Department of Plastic Surgery, The Sahlgrenska Academy, Gothenburg University, Gothenburg, Sweden; 3 Department of Plastic and Reconstructive Surgery, Sahlgrenska University Hospital, SE-413 45 Gothenburg, Sweden

**Keywords:** BREAST-Q, PROM, Breast reduction, Mastopexy, Normative values, Breast hypertrophy

## Abstract

**Background:**

Norm values for patient reported outcomes, that is knowledge about how the general population of women rate their breast-related satisfaction and quality of life, are necessary to interpret the meaning of scores. The aims of this study were to create Swedish normative values for the BREAST-Q reduction/mastopexy module and to describe what healthy women are most satisfied/dissatisfied with regarding their breasts.

**Methods:**

A random sample of 400 women aged 18-80, currently living in Region Västra Götaland, were sent BREAST-Q reduction/mastopexy. Descriptive data are presented.

**Results:**

One hundred and forty-six women answered the questionnaire (36.5%). Mean total scores ranged from 48 to 78. No clear changes in scores could be seen with age and women with a high BMI seem to be less satisfied with their breasts. The participants were most satisfied with the appearance of the breasts when dressed, the appearance in the mirror dressed, the shape of the breasts with bra, and symmetry of size and most dissatisfied with appearance in the mirror naked and the shape of the breasts without a bra. Thirty to forty-five per cent of healthy women never or almost never feel sexually attractive. Among physical symptoms often described in breast hypertrophy, the most common among healthy women were lack of energy, pain in the neck, arms and shoulders, headache and difficulty performing intense physical activity.

**Conclusion:**

The norms for BREAST-Q reduction/mastopexy add another piece to the puzzle to what constitutes normal breast satisfaction and how surgical outcomes can be evaluated.

**Level of Evidence IV:**

This journal requires that authors assign a level of evidence to each article. For a full description of these Evidence-Based Medicine ratings, please refer to the Table of Contents or the online Instructions to Authors  www.springer.com/00266.

## Introduction

Breast hypertrophy may affect a woman’s life both physically, with pain, intertrigo, inability to exercise and to perform certain activities, and psychosocially with body image problems, low self-esteem, and sexual problems [1]. Given the type of problems breast hypertrophy give rise to, the effect of a breast reduction is often evaluated with patient reported outcome measurements (PROMs) [1]. Several PROMs have been developed for breast hypertrophy, of which BREAST-Q is the most widely used [1, 2]. PROMs consistently show that patients are more satisfied with their breasts and have a higher quality of life (QoL) after a breast reduction [3–5]. This effect can be seen both in breast reductions performed in the public health care system [3–5] as well as in privately financed aesthetic surgery [6, 7], which makes it difficult to use improvement in patient satisfaction and QoL as indicators that the surgery should be performed in the publicly financed health care system [1]. One aspect to consider when deciding if an operation should be rationed or not could be to evaluate if the woman’s breast related satisfaction and QoL is within statistical normality [8]. Such an evaluation requires norm values for PROMs, that is knowledge about how the general population of women rate their breast-related satisfaction and QoL [9].

Previously, two normative populations, one North American [10] and one Dutch [11], have been described for the BREAST-Q reduction/mastopexy module (Table 1). The North American norms [10] were created by sending the questionnaire to 121 688 members of The Army of Women, which is an online community promoting breast cancer research. Respondents had to be > 18 years without a history of breast cancer or breast surgery. Data collection was automatically stopped when 1200 women had answered the questionnaire. The exact response rate is unknown as it is not known how many of the 121688 approached women were eligible for participation. The Dutch norms [11] were created by letting a randomly chosen sample of women registered in the municipal base administration in the north of the Netherlands answer the questionnaire. The response rate was 28 per cent (1334/4623). Previous experiences of creating norm populations for the BREAST-Q reconstruction module have indicated that there could be cultural variations in breast-related satisfaction and QoL between different populations [12] and that population specific normative values therefore are needed in order for them to be useful in a scientific context.

The primary aim of this study was to create Swedish normative values for the BREAST-Q reduction/mastopexy module. Secondary aims were to describe what healthy women are most satisfied/dissatisfied with regarding their breasts and to compare the norms with BREAST-Q values of patients with breast hypertrophy.

## Materials and Methods

### Study Protocol and Ethics

This is a cross-sectional cohort study pre-registered on ClinicalTrials.Gov, identifier NCT04526561. The Regional Ethical Committee of Gothenburg reviewed and approved the study (254-18). The procedure followed were in accordance with the Declaration of Helsinki.

### Setting, Participants, and Data Collection

Region Västra Götaland is situated in Western Sweden and has about 1.7 million inhabitants. The principal city is Gothenburg. A random sample of 400 women aged 18-80 years, currently living in Region Västra Götaland, was made by *Statens personadressregister, SPAR,* which includes all residents in Sweden. The only exclusion criterium was inability to understand Swedish. Patients were not excluded due to previous breast surgery or breast disease, as these patients are part of the general population. The women were sent information on the study, the BREAST-Q domains described in the next section, and a stamped return envelope. Two remainders were sent after four and eight weeks. The participants were asked to state their age, weight, and height. A previous study [3], in Region Västra Götaland, on patients with breast hypertrophy (n=156), that is a breast volume of > 800 ml with a normal body mass index (BMI) (<25 m/kg^2^) [3] were used as controls (Table 1).

### BREAST-Q

The items and domains of BREAST-Q were developed with qualitative technique and literature review and field-tested in North America [13, 14]. The questionnaire has undergone psychometric testing and been translated to Swedish. The BREAST-Q reduction/mastopexy module comprises the following pre-operative domains: Satisfaction with breasts (11 items), Psychosocial well-being (9 items), Sexual well-being (6 items), and Physical well-being chest (14 items). The items are rated on a Likert scale from 1 to 4 or 5. A raw summed domain score is calculated and transformed to Rasch logits and subsequently transformed to a standardised domain score between 0 and 100. A higher score indicates a better outcome/higher satisfaction. Use of BREAST-Q, authored by Drs. Klassen, Pusic and Cano, was made under license from Memorial Sloan Kettering Cancer Center, New York, USA. The questionnaire is available from https://qportfolio.org/breast-q/reductionmastopexy/.

### Statistics

BREAST-Q data and missing values were treated as described in the BREAST-Q manual [15]. QScore^TM^ was used to calculated summary scores for the domains. Descriptive data were given as median, mean, range, and standard deviation (SD) for the domain scores, and frequencies for the categorical item scores. Histograms were drawn to illustrate data distribution. Differences between the norms and patients with breast hypertrophy were tested with two-sided single sample t-test. Differences between different age and BMI groups were tested with one-way ANOVA. A p-value of <0.05 was considered to indicate a statistically significant difference. Statistical analyses were preformed using Microsoft Excel version 16.58 (Redmond, Washington, USA) and SPSS^®^ version 26.0.0.0 (IBM, Armonk, New York, USA).

## Results

**Fig. 1.**
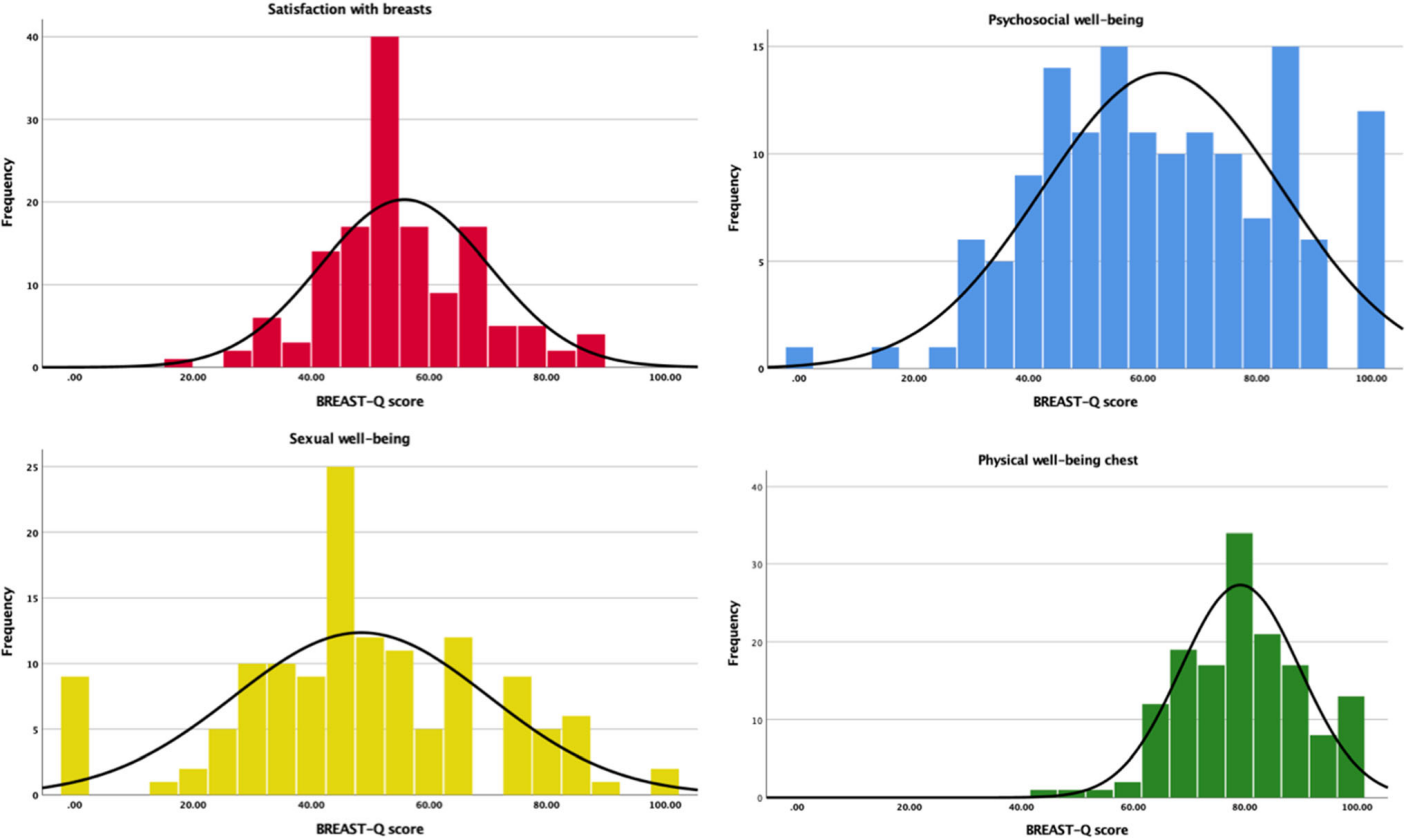
Histograms of normative Swedish BREAST-Q scores for the different domains

From December 2021 to March 2022, 146 women answered the questionnaire giving a response rate of 36.5 per cent (146/400). All 146 participants answered the domains Satisfaction with breasts, Psychosocial well-being, and Physical well-being chest, whereas 134 participants (33.5%) answered the Sexual well-being domain. The mean age of the cohort was 53 years (SD 17) and mean body mass index (BMI) was 25 kg/m^2^ (SD 4). The normative total scores for the different BREAST-Q domains are given in Table 1 and Fig. 1. Mean total scores ranged from 48 to 78 and SDs from 14 to 25. It seems that middle-aged and older women were more prone to answer the questionnaire than young women. No clear difference in scores could be seen with age for Satisfaction with breasts and Psychosocial well-being, whereas middle-aged women seemed more satisfied with their sexual well-being and middle-aged and women over >70 years of age seemed more satisfied with their physical well-being (Table 2). Women with a high BMI seem to be less satisfied with their physical well-being (Table 3). The domain scores were similar to the US norms, slightly lower than the Dutch norms, and considerably higher than the scores from patients with breast hypertrophy (Table 1).

**Table 1:** Swedish norms, Swedish controls, and US norms

	Satisfaction with breasts Mean (SD) Median (range)	Psychosocial well-being Mean (SD) Median (range)	Sexual well-being Mean (SD) Median (range)	Physical well-being chest Mean (SD) Median (range)
Swedish norms of the present study (*n*=146)	56 (15) 54 (19-100)	63 (22) 62 (0-100)	48 (25) 46 (0-100)	78 (14) 79 (44-100)
Swedish controls with breast hypertrophy [3] (*n*=156)	24 (15) 23 (0-100)	38 (17) 38 (0-87)	40 (23) 38 (0-100)	54 (12) 56 (6-83)
Swedish norms vs. Swedish controls (One-sample t-test)	*p*<0.001	*p*<0.001	*p*<0.001	*p*<0.001
US norms [10] (*n*=1205)	57 (16) NR	68 (19) NR	55 (19) NR	76 (11) NR
Dutch norms [11] (*n*=1334)	68 (19) NR	72 (17) NR	58 (19) NR	80 (14) NR

*NR*: Not reported, *SD*: standard deviation

**Table 2:** Swedish BREAST-Q norms for different age groups

Age (n=145)	Satisfaction with breasts Mean (SD)	Psychosocial well-being Mean (SD)	Sexual well-being Mean (SD)	Physical well-being chest Mean (SD)
18-30 (*n*=17)	59 (17)	59 (27)	49 (19)	79 (7.8)
31-40 (*n*=20)	52 (14)	61 (26)	49 (23)	79 (9.8)
41-50 (n=21)	58 (15)	64 (19)	55 (18)	83 (11)
51-60 (n=30)	59 (14)	70 (20)	54 (24)	79 (11)
61-70 (n=33)	54 (13)	59 (19)	43 (25)	75 (12)
>70 (n=24)	52 (11)	63 (19)	39 (25)	82 (11)
Differences between different age groups (ANOVA)	*p*=0.25	*p*=0.31	*p*=0.041	*p*=0.035

*SD*: Standard deviation

**Table 3.** Swedish BREAST-Q norms for different BMI groups

BMI (n=144)	Satisfaction with breasts Mean (SD)	Psychosocial well-being Mean (SD)	Sexual well-being Mean (SD)	Physical well-being chest Mean (SD)
<18.5 (*n*=2) 1	46 (21)	67 (6.4)	43 (19)	83 (5.7)
18.5-25 (*n*=84) 2	56 (13)	65 (21)	49 (24)	81 (11)
25.1-30 (*n*=42) 3	55 (16)	61 (22)	50 (24)	79 (9.5)
30.1-35 (*n*=11) 4	58 (13)	59 (19)	51 (26)	70 (10)
>35.1 (*n*=5) 5	45 (17)	64 (29)	28 (21)	77 (14)
Differences between different BMI groups (ANOVA)	*p*=0.80	*p*=0.92	*p*=0.26	*p*=0.03

*SD*: Standard deviation

**Fig. 2.**
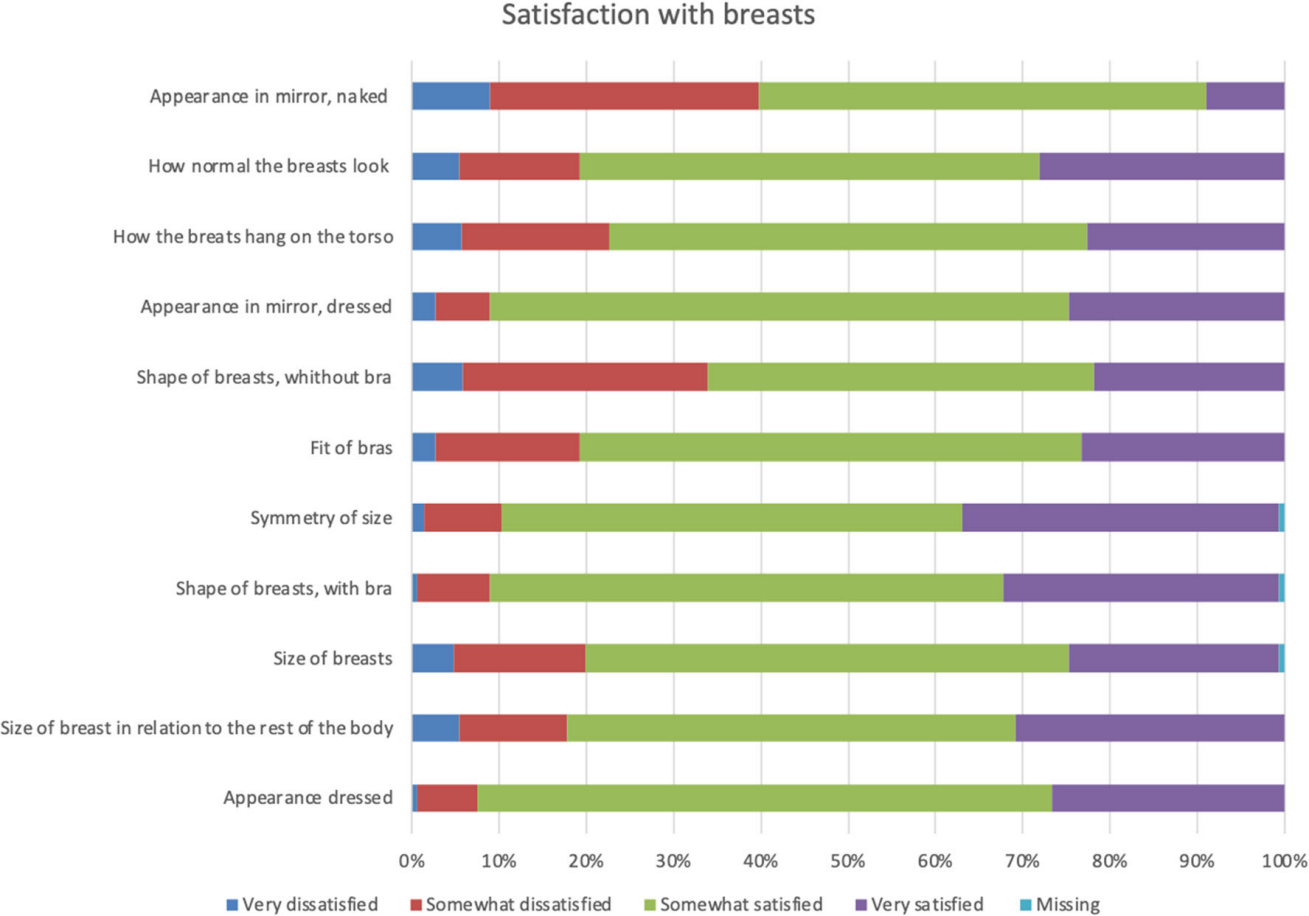
Frequencies of answers of different BREAST-Q items for the domain Satisfaction with breasts

**Fig. 3.**
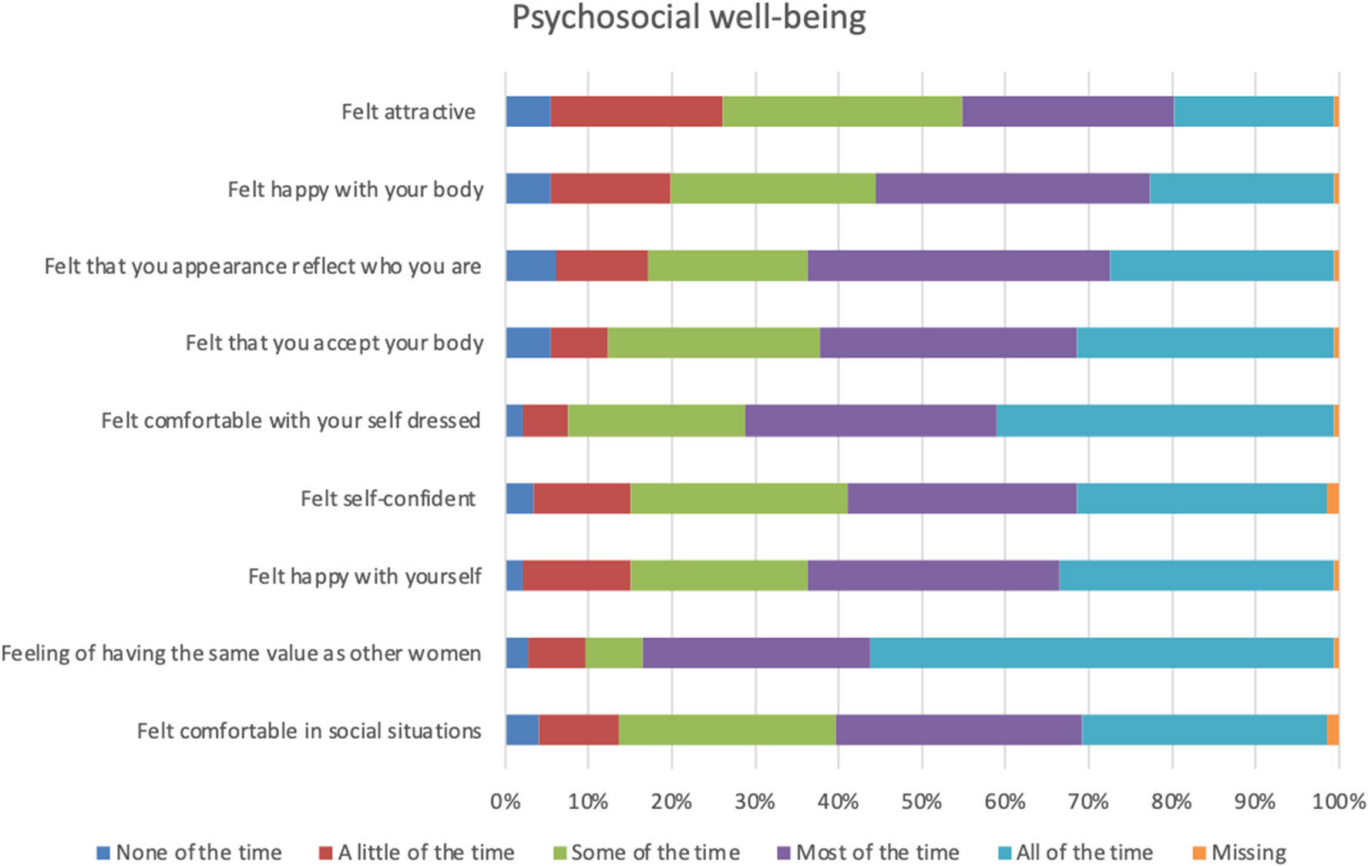
Frequencies of answers of different BREAST-Q items for the domain Psychosocial well-being

**Fig. 4.**
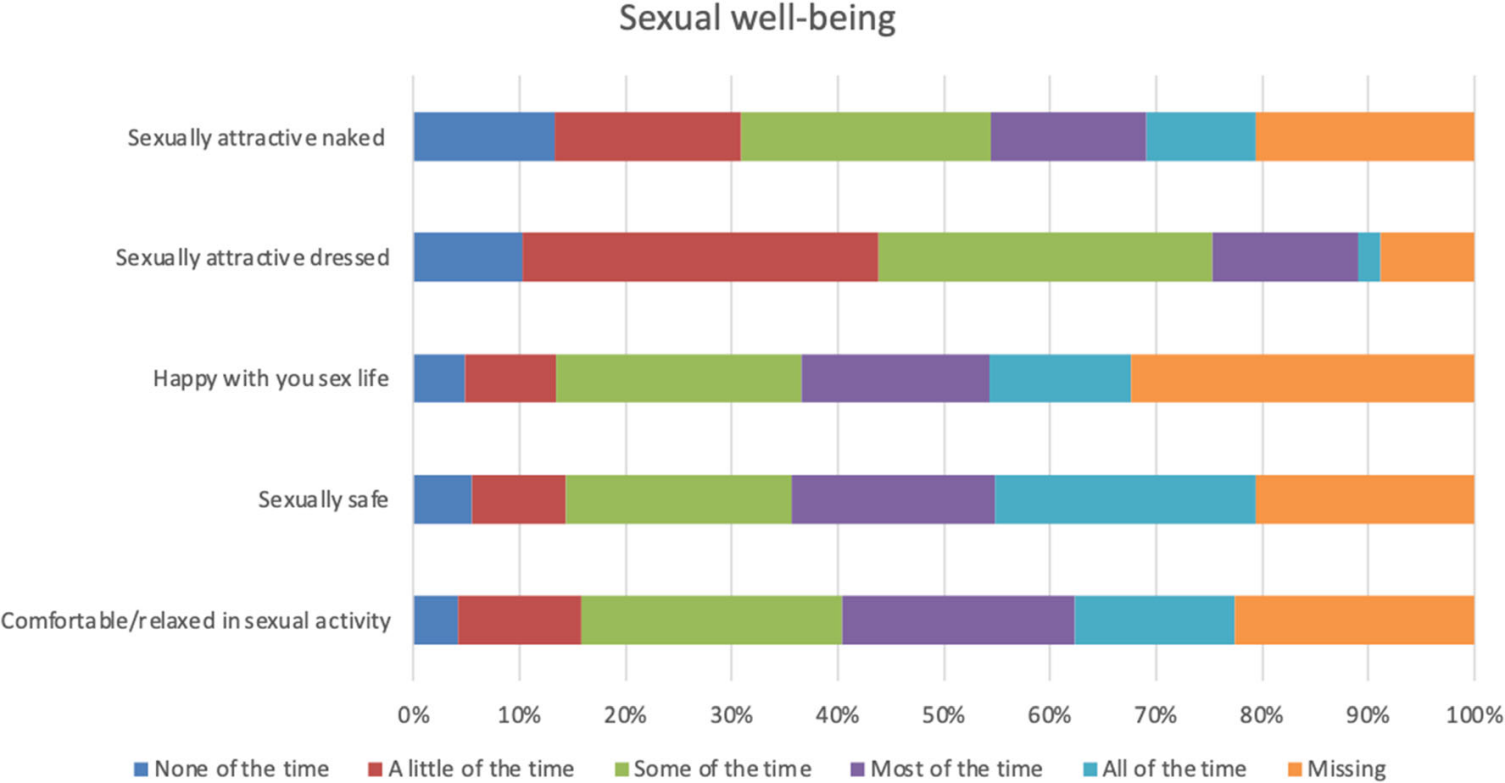
Frequencies of answers of different BREAST-Q items for the domain Sexual well-being

**Fig. 5.**
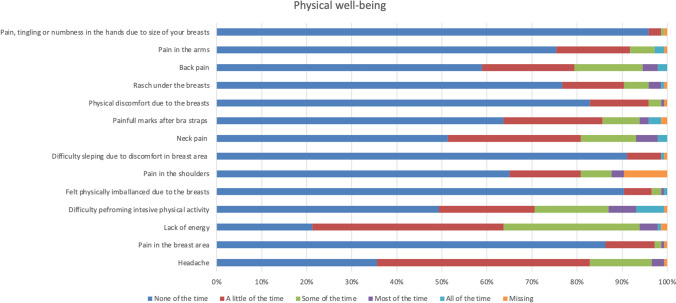
Frequencies of answers of different BREAST-Q items for the domain Physical well-being chest

The participants were most satisfied with the appearance when dressed, the appearance in the mirror dressed, the shape of the breasts with a bra, and symmetry of size with which 90 per cent or more were somewhat or very satisfied (Fig. 2). They were most dissatisfied with the appearance in the mirror naked and the shape of the breasts without a bra, with which 40 and 35 per cent, respectively, were very or somewhat dissatisfied with (Fig. 2). More than half of the women were happy with their body, and thought that their appearance reflects who they are, and felt comfortable with themselves when dressed, self-confident, happy with themselves, comfortable in social situations, that they have the same value as other women and, accept their bodies most or all of the time (Fig. 3). About one fourth of the healthy women felt attractive none or a little of the time, one fifth were happy with their body none or a little of the time, and almost one fifth never or almost never felt that their body reflects who they are (Fig. 4). Thirty to forty-five per cent of healthy women never or almost never felt sexually attractive (Fig. 4). Among the physical symptoms often described among women with breast hypertrophy, the most common among healthy women were lack of energy, pain in the neck, arms and shoulders, headache and difficulty performing intense physical activity (Fig. 5).

## Discussion

This study presents the first normative data for BREAST-Q reduction/mastopexy in Scandinavia. It was generated in a randomly selected sample of the general population of Region Västra Götaland, Sweden.

### Considerations Regarding the Methodology – Strengths and Weaknesses

It is challenging to generate norms from a truly representative sample of the general population. In this study, a sample was randomly selected among all women between 18 and 80 years of age that are residents in Region Västra Götaland. Therefore, the sample itself should be representative of the general population. However, the incomplete response rate might have affected the representativeness. Indeed, the average age of the sample is about ten years higher than the average age in Region Västra Götaland according to *Statistics Sweden*. To preserve participants’ privacy, we have previously sent the questionnaire to a 1000 randomly selected women without coding and received 157 answers. Therefore, a new attempt was made, with a new sample of randomly selected women created by SPAR. In the current investigation, the questionnaires were coded and therefor remainders could be sent. Although two remainders were sent to every woman, and demographic data requested were limited to age, height and weight, only 36.5 per cent of the women replied. Women who have had previous breast surgery, due to disease or for aesthetic reasons, were not excluded from the present study, which might have affected the result. Women who have had surgery were not excluded as they do indeed constitute part of a sample of women from the general population. Although, statistical comparisons were performed between different age groups and different BMI groups, the results should be interpreted with caution as the sample was very small in some age/BMI groups. A strength of the present study is that the questionnaire was sent to a truly random sample of the general population, albeit with a less than perfect response rate; although, representativeness cannot be guaranteed.

### Considerations Regarding the Results

The study suggests that the Swedish norms are similar to the published US [10] and lower than the Dutch norms [11]. Previously, it has been suggested that there might be a considerable cultural difference in breast satisfaction norms between different populations [12] and that theory is strengthened by the present results. The finding that BMI seems to affect the satisfaction with breasts corroborate previous studies indicating that a higher BMI leads to a lower satisfaction with the breasts [10, 12]. However, this finding should be interpreted with caution as BREAST-Q reduction/mastopexy is specifically designed to detect quality of life effects of large volume breasts and breasts usually grow in size with an increasing BMI. Moreover, a high *BMI* per se might lead to a lower body satisfaction [16].

In Western countries, breast operations, including both augmentations, reductions, and mastopexies, are among the most common aesthetic operations performed [17], suggesting that many healthy women are dissatisfied with different aspects of their breasts. This was clearly reflected in the present study, where the average value was close to half of the full score of 100 (Table 1). The women were clearly more satisfied when dressed than when naked (Fig. 1). BREAST-Q reduction/mastopexy should be able to clearly discriminate between healthy women and women with hypertrophic and/or ptotic breasts. Compared with a previous study on breast hypertrophy in Region Västra Götaland*,* the normative values were considerably higher in all domains (Table 1). Similarly, US studies on patients with beast hypertrophy have revealed lower scores (mean 20 to 46) than the norms [5, 18]. Nonetheless, these studies were performed in patients who had actively requested breast surgery and they were therefore *per definition* dissatisfied with their breasts. This leads to the question whether QoL instruments can be used to select which patients should be operated on in the public health care system. More studies are needed to investigate how much the scores should deviate from statistical normality to warrant a rationed operation. Moreover, if a woman has a low BREAST-Q score, which she probably has if she is requesting breast surgery, it is likely that her score will improve considerably with surgery due to the phenomenon of *regression to the mean* [19]. *Regression to the mean* describes that an extreme circumstance, in this case a low satisfaction with the breasts, probably is followed by a more typical situation, that is a mean BREAST-Q score. Following that, all women with a very low satisfaction with their breast would probably score much higher post-operatively, even if a significant clinical change in satisfaction has not occurred. This complicates the evaluation of outcomes of surgery and the value of a particular intervention in relation to other inventions, in case of breast hypertrophy for example weight loss interventions. More studies are needed on BREAST-Q to make it an effective primary outcome measure in clinical trials.

## Conclusion

The norms for BREAST-Q reduction/mastopexy add another piece to the puzzle to what constitutes normal breast satisfaction and how surgical outcomes can be evaluated. Further studies are needed regarding how knowledge about statistical normality can be used to decide which patients should be operated in the publicly funded health care system.
